# CO_2_ and Acidification of Low-Salt Brine Promote Some Yeasts and Penalize Bacteria in Naturally Brined Black Table Olive Fermentation

**DOI:** 10.3390/foods14234062

**Published:** 2025-11-27

**Authors:** Biagi Angelo Zullo, Gino Ciafardini

**Affiliations:** Department of Agricultural, Environmental and Food Sciences, University of Molise, Via de Sanctis, I-86100 Campobasso, Italy

**Keywords:** antimicrobial activity, black table olive, CO_2_, fermentation, low-salt brine

## Abstract

Naturally fermented black table olives are typically processed in brine with a high NaCl content. Since salt is responsible for several cardiovascular problems, methods are needed to reduce the salt (NaCl) content in the olive flesh. In this study, we investigated the natural fermentation of healthy and damaged black table olives marinated in acidified low-salt brine under slightly pressurized CO_2_ (spCO_2_) conditions. Tests performed with healthy black table olives of the Leccino cultivar showed the presence of yeasts and the absence of bacteria and molds in the brine during the entire fermentation period. Among the yeasts, *Saccharomyces cerevisiae* prevailed, especially at the end of the fermentation period. Black table olives damaged by the olive fruit fly *Bactrocera oleae* (Rossi) were contaminated by various microorganisms, including enterobacteria and lactic acid bacteria, recorded mainly in fruit fly larvae. During fermentation with acidified low-salt brines under spCO_2_, enterobacteria did not survive at the beginning of incubation, whereas the lactic acid *Leuconostoc mesenteroides* identified at the beginning of fermentation disappeared after 2 months of incubation. Among the yeasts that survived during the incubation, *S. cerevisiae* clearly prevailed. All results confirmed the antimicrobial activity of acidified low-salt brine in the presence of spCO_2_. This technology may offer a potentially safer method for production of low-salt olives.

## 1. Introduction

Table olives, produced from olive trees (*Olea europea* L.), represent an important fermented plant product in the Mediterranean diet due to their high content of nutrients and bioactive compounds beneficial to human health [1]. They constitute a noteworthy economic factor for the Mediterranean countries since both their production and consumption are exponentially increasing year by year, worldwide [2]. Before consumption, table olives must be processed to reduce their content of oleuropein and its aglycones, which impart a bitter taste to the fruit. Table olives are prepared through three main methods: the Spanish process, in which green olives are treated with lye; the Californian process, which incorporates lye treatment and air oxidation and the natural-style method where the fruits are brined without going through a NaOH treatment and left to ferment until they lose their bitterness at least partially [3,4]. Each method of debittering produces a different style of table olives with a unique texture and chemical, microbial and sensorial profiles. The traditional natural processing of black table olives is carried out by marinating the fruits for 8–12 months in brine with 8–12% (*w v*^−1^) NaCl in 160–200 L polyvinyl chloride (PVC) barrels or in 20 tons stainless steel silos. The presence of salt in the brine, in synergy with the low pH and the phenolic compounds of the fruit, has a “hurdle effect” on many microorganisms that are harmful to the quality of the product and the health of consumers. The quality of the product can be jeopardized by the high presence of some yeast species responsible for spoilage, while several bacterial species, including *Escherichia coli*, *Clostridium botulinum* and *Listeria monocytogenes*, are highly dangerous for humans and can even cause death [5,6,7]. Strict adherence to Good Manufacturing Practices (GMPs), ensuring sufficient salt concentration (>5%) and a final pH below 4.3 allows some of these bacteria to be controlled [8]. However, although the nutritional properties of the naturally brined table olives are well known, their consumption can generally be limited by their high salt content. Salt consumption is one of the major concerns of the population and saltiness is one of the attributes included in the sensory evaluation sheet of table olives [9]. According to the World Health Organization (WHO) [10], the recommended daily salt intake should be reduced to <5 g/day of salt for adults and the European Community reference intake has been set at 2.4 g of NaCl per day [11]. Low salt intake helps reduce blood pressure and the risk of cardiovascular disease [12]. The reduction of NaCl content in the brine during olive fermentation and in the final product has attracted the attention of the scientific community in the last decade. Some studies have focused on the use of salt mix mixtures during the fermentation process and storage of olives in brine [13,14,15], others instead have concerned the desalination process of the product before packaging [16]. However, for a healthy risk prevention, the high salt levels normally used during fermentation can hardly be reduced with the current state-of-the-art technology for naturally fermented table olives, since the low salt content does not always guarantee the correct safety of the product. In low-salt brines, if other physicochemical parameters such as pH are also used, it is possible to increase the number of factors that have a “hurdle effect” on the growth of spoilage microorganisms. In some of our previous studies, we demonstrated, for the first time, that a slightly pressurized carbon dioxide (spCO_2_) less than 1 Mpa, can inhibit or reduce microbial growth in the brine of naturally fermented black table olives with low pH and reduced NaCl concentration. This effect seems more evident on bacteria rather than on yeasts, which develop differently depending on the species and the chemical–physical characteristics of the brine. As reported in other study, the CO_2_ dissolved in the brine as carbonic acid penetrates the microbial cells inhibiting their growth [17]. Considering that for the reasons stated above many bacterial and fungal species are unwanted, interestingly, the tests carried out at pilot plant scale indicated that a spCO_2_ of 1 bar is able to inhibit the growth of bacteria and molds in a brine with reduced salt concentrations [18]. Other research carried out on the fermentation of black table olives with a reduced NaCl content, confirmed that the growth of microorganisms in the brine is selectively inhibited by spCO_2_, low pH, and salt [19]. On the basis of these results it seems that the immobilization in the brine of the CO_2_ released by the fermentation process and by the respiration of the fruits, in association with salt, the low pH and the polar phenols released by the fruit has a “hurdle effect” on the survival of many bacteria. The production of table olives with low salt content, through the sequestration of CO_2_ produced by the fermentation of the same olives in brine, also presents other advantages such as the limitation of gaseous emissions into the environment and the inhibition of the appearance of microbial biofilm on the surface of the brine in the barrels, due to the penetration of external air [20]. However, studies on the use of spCO_2_ in the production of naturally brined black table olives, although promising, are still limited. Studies on the use of spCO_2_ have been conducted on healthy olives, while the microbial succession in the fermentation of fly-damaged olives under spCO_2_, as well as the investigation of *L. mesenteroides* survival, remain unexplored. This study evaluated the effect of spCO_2_ and acidification on microbial community of black table olives under reduced salt conditions.

## 2. Materials and Methods

The olives of cv. Leccino (*Olea europaea* L.), characterized by a maturation index (MI) of four, were established according to Uceda and Frìas [21] and came from two different batches. The first batch consisted of healthy, good-quality olives, free from attacks by the insect Tephritidae *Bactrocera oleae* (Rossi) known as the olive fruit fly. Before undergoing fermentation tests under spCO_2_ conditions, some of them were used without any pre-treatment, while the remainder were washed with potable water to reduce initial microbial contamination. In contrast, the olives from the second batch were more contaminated by microorganisms since they were physically damaged by the olive fruit fly *B. oleae*, which had produced tunnels in 10% of the fruits. Olive healthiness was measured by inspecting the pulp integrity of 100 fruits picked at random from different batches of olives. Furthermore, the same olives were used for the tests without any pre-treatment with potable water.

### 2.1. Tests with Healthy Table Olives

After removing the leaves and foreign bodies, the olives from the first batch were divided into two fractions of approximately 15 kg each. The first was washed with tap water and used for the tests, while the second was used without undergoing any washing with potable water.

#### 2.1.1. Fermentation Trials with Washed Healthy Table Olives

A total of 5 kg of good-quality Leccino olives from the first batch were placed in a 20 L stainless steel tank and subjected to two wash cycles with tap water. Washing was performed by agitating the fruit suspended in the aqueous mass for 10 min. At the end of each cycle, the wash water was discarded and replaced with clean water. After washing, the olives were distributed into commercial glass jars with a nominal volume of 314 mL equipped with a metal lid with a “click” pressure indicator device capable of maintaining the pressure at 1 bar. Each glass jar contained approximately 190 g of olives covered with 120 mL of brine containing 6% (*w v*^−1^) NaCl and 0.5% (*w v*^−1^) citric acid. Twenty tightly closed glass jars were prepared and stored at 15–17 °C for 8 months. During incubation, three glass jars for each sample were randomly taken and analyzed at 7, 60, 120, 180, and 240 days. Glass jars opened after analysis were discarded. Fermentation took place under spCO_2_ conditions starting on the first day of incubation. During the first six months of incubation, the brine was subjected to microbiological and chemical analysis, while at the end of the trial the same analyses were performed on both the brine and the flesh.

#### 2.1.2. Fermentation Trials with Unwashed Healthy Table Olives

A total of 15 kg of Leccino olives from the first batch were not washed with tap water in order to preserve the original composition of the fruit’s microbiota. The olives were placed directly into glass jars with a nominal volume of 314 mL equipped with a metal lid with a “click” pressure indicator device. The test was accomplished similarly to the previous one, using the same quantity of olives per jar and the same volume of brine containing 6% (*w v*^−1^) NaCl and 0.5% (*w v*^−1^) citric acid. Seventy-five jars were prepared and stored in the dark at 15–17 °C for 30 days. At each sampling, three jars were taken randomly and subjected to microbiological and chemical analysis. The samples were analyzed daily and discarded after each sampling.

### 2.2. Tests with Damaged Table Olives

A total of 15 kg of Leccino olives from the second batch damaged by the fruit fly *B. oleae* were harvested at the same ripening stage as in the previous trials. After removing leaves and foreign bodies, the olives were used for the trial without washing them with potable water, in order to maintain the composition of their original microbiota. At the same time, the microbial concentration in the brine used for the trials was improved by inoculating them with a starter produced separately as described below. The trial was accomplished similarly to the previous trials, placing approximately 190 g of olives and 120 mL of brine in glass jars with a nominal volume of 314 mL, equipped with a metal lid with a “click” pressure indicator device. The brine contained 6% (*w v*^−1^) NaCl and 0.5% (*w v*^−1^) citric acid. At the time of use, 8 L of brine was mixed with 1.5 L of starter and then used immediately to prepare fifty-four tightly closed glass jars with fruits and stored for 180 days at 15–17 °C. During incubation, three glass jars samples were randomly removed and the inoculated brine was subjected to microbiological and chemical analysis. Glass jars opened were discarded after each analysis.

#### Starter Preparation

Healthy washed Leccino black table olives from the first batch were placed in a 5 L polyethylene terephthalate (PET) demijohn bottle equipped with a large self-screw cap, containing 2.6 kg of olives covered with 2.4 L of brine containing 6% (*w v*^−1^) NaCl, not acidified with citric acid. The demijohn bottle was hermetically sealed with the screw cap and incubated under spCO_2_ self-produced by the respiratory activity of the fruit and fermentation process. Incubation under spCO_2_ occurred for 7 days at 15–17 °C. At the end of the incubation, the brine was analyzed microbiologically and then used to inoculate the brine from the previous trial, using the doses reported above.

### 2.3. Microbiological Analysis

Microbiological analysis was performed using both brine and olives to quantify the main microbial group (bacteria, yeasts, and molds) involved in the black table olive fermentation. Among the bacteria, total aerobic bacteria (TAB), total anaerobic bacteria (TANB), and enterobacteria were evaluated. The brine was analyzed directly without undergoing any pretreatment. However, the olives were treated as follows before being subjected to microbiological analysis. The fruits were manually pitted with a metal device previously sterilized at 120 °C for 20 min. Subsequently, the flesh collected in a sterile beaker was homogenized in the presence of sterile Ringer’s solution (0.9% NaCl, *w v*^−1^) following the procedure described by Zullo and Ciafardini [22]. Brine and flesh samples were serially diluted by a factor of 10 in sterile Ringer’s solution and spread on specific agar media, as described below. The TAB were enumerated after 24 h of incubation at 30 °C on nutrient agar (NA) (CM 0003, Oxoid, Basingstoke, UK) supplemented with 0.05% (*w v*^−1^) cycloheximide (Sigma-Aldrich, St. Louis, MO, USA). The TANB were enumerated on peptone yeast-extract glucose agar (PYGA) medium [23]. The number of bacterial colonies was recorded after 72 h of incubation at 30 °C under anaerobiosis (AnaeroGen and AnaeroJar, Oxoid). Violet Red Bile Glucose (VRBG; Biolife, Milan, Italy) agar was used for the enumeration of enterobacteriaceae. After the inoculation, the plates were incubated aerobically at 37 °C for 24 h and the colonies were recorded. The number of total yeasts was estimated on Malt, Yeast, Glucose, Peptone (MYGP) agar medium [18] supplemented with chloramphenicol (0.1 g L^−1^). The colonies were recorded after 5 days of incubation at 30 °C. The total molds were evaluated after 7 days of incubation at 28 °C on Malt Extract (ME) agar medium with the following composition, 20 g malt extract (BBL, Cockeysville, MD, USA), 1 g casein bacto tryptone (BD, Sparks, MD, USA), 20 g D-glucose (Merck, Darmstadt, Germany), 15 g agar (Sigma-Aldrich, Milan, Italy), and 1000 mL distilled water. All the microbiological analysis were repeated 3 times for each sample and the results were expressed as Log values of Colony Forming Units per mL of brine or g flesh (Log CFU mL^−1^/g^−1^). The detection limit was established as 10 CFU mL^−1^/g^−1^.

### 2.4. Total Aerobic Bacteria Identification

Total aerobic bacteria identification tests involved only TAB, as only they grew in one of the fermentation trials described above. Approximately 200 bacterial colonies from the brine of black table olives processed under spCO_2_ conditions were used to prepare some Master cultures and employed in the identification tests. Identification was performed based on the physiological characteristics of the bacterial isolates associated with analyses performed using the matrix-assisted laser desorption/ionization time of flight mass spectrometry (MALDI-TOF MS). The main physiological characteristics were ascertained as follows. Cell shape was observed with a light microscope equipped with phase contrast. Gram staining was performed with the Gram staining kit for microscopy (Sigma-Aldrich). The production of catalase was demonstrated by placing a drop of 3% (*v v*^−1^) H_2_O_2_ on the colonies, whereas tryptophan medium was used for the production of ammonium [24]. Durham tubes were used to detect gas produced by bacteria. These special tubes (7.25 × 50 mm) were placed upside down in the test tubes containing the bacterial cultures in Nutrient Broth (Oxoid). The gas produced by the bacteria gets trapped in the Durham tube, forming a visible bubble. The production of dextran was detected in agar medium with sucrose according to Sarwat et al. [25]. Litmus milk coagulation was tested by inoculating an overnight bacterial culture into a medium consisting of powdered milk (100 g L^−1^), bromophenol blue (0.75 g L^−1^), glucose (20 g L^−1^), and yeast extract (5 g L^−1^). Coagulation was visually evaluated in the growth medium after 40 h incubation at 30 °C. The nitrate reduction, bacterial growth at 37 °C and 45 °C as well as the growth in presence of 4% (*w v*^−1^) and 6.5% (*w v*^−1^) NaCl were done as reported by Ciafardini and Zullo [26]. Identification with MALDI-TOF MS was performed on 20 bacterial isolates taken randomly from among the 200 colonies of the master cultures. Briefly, the TAB isolates were grown on NA medium supplemented with 0.02% (*w v*^−1^) cycloheximide (Sigma-Aldrich) for 72 h at 30 °C. A small portion of bacterial colony was subsequently analyzed using a Bruker Microflex LT MS machine (Bruker Daltonics, Manning Park, Billerica, MA, USA). Rapid on-plate formic acid (FA) treatment, followed by the application of α-cyano-4-hydroxycinnamic acid (HCCA), was used for protein extraction from bacterial cells, according to the manufacturer’s instructions. The mass spectra profiles were assessed, visualized, and analyzed/compared to reference spectra from the Bruker Biotyper version 3.1 (build 65) database using bruker flexControl 3.4 software.

### 2.5. Yeast Biodiversity

The yeast colonies obtained from the microbiological analysis of the brines during fermentation and olives after fermentation, were used to prepare master cultures established in Petri dishes with MYGP agar medium. After 3 days of incubation at 30 °C, the master cultures with 100 colonies each were replicated on CHROMagar Candida medium (BBL, Cod. 4354093, Heidelberg, Germany) and tested as described by Tornai-Lehoczki et al. [27]. The yeasts were analyzed under a light microscope to ascertain the presence of pseudohyphae and cell shape. The main characteristics suitable for distinguishing the different chromogenic yeast groups were the cell shape, the presence of pseudohyphae, the morphology, and color of the colony. From each chromogenic group of each analyzed sample, 5 yeast isolates were randomly selected and subjected to molecular identification at species level using the previous procedures described by Ciafardini and Zullo [20].

### 2.6. Physicochemical Parameters

Physicochemical analysis was performed on both the brine and the fruits during fermentation and at the end of the incubation of the experiments reported above, respectively. The analyses involved determination of pH, NaCl concentration, free CO_2_, and total polar phenols content. The brine was analyzed directly without undergoing any pretreatment, whereas the olives were treated as follows before being subjected to chemical analysis. The fruits were manually pitted with a metal device. Subsequently, 10 g of pitted pulp was placed in a beaker and 50 mL of a methanol-distilled water (80:20, *v v*^−1^) mixture was added. After 2 min of extraction (Turrax model T25; IKA, Milan, Italy), the paste was centrifuged at 9000× *g* for 10 min using a Hettich centrifuge (Centrifuge model Universal 32, Hettich Instruments, Tüttlingen, Germany). The phenolic content of the extract thus obtained was evaluated. The concentration of total polar phenols in the brine and in the fruits was evaluated using the Folin-Ciocalteu’s phenol colorimetric method, as described by Ciafardini and Zullo [20]. The concentration of total polar phenols was expressed as mg of caffeic acid equivalent per mL of brine or g flesh. The chemical analysis was repeated three times for each sample. To evaluate the pH of the olives, 2 g of pitted pulp was placed in a 25 mL beaker and 10 mL of distilled water was added. After 2 min of homogenization (Turrax model T25), the oily paste was used for pH determination. For both the brine and the olives, the pH was determined via pH meter using an In Lab Routine Probe (Mettler, Toledo, OH, USA). Three measurements were performed on each sample. The NaCl content in brine and flesh was evaluated using Mohr’s method. A total of 1 mL of brine or the extract sample described for the pH assessment was first diluted with 50 mL of distilled water and then titrated with 0.1 N AgNO_3_ using K_2_CrO_4_ as the indicator [3]. The pH of the sample was adjusted to 6.5 using 1 N NaOH solution, before titration with AgNO_3_ [22]. The concentration of free CO_2_ was evaluated only in the brine. The CO_2_ concentration dissolved in the brine was evaluated by titration of the brine using a 0.0454 N solution of anhydrous Na_2_CO_3_ as described by Zullo and Ciafardini [22]. The concentration of CO_2_ dissolved in the brine was expressed as grams of free CO_2_ per liter of brine. Each chemical analysis was repeated three times.

### 2.7. Evaluation of Resistance of Leuconostoc Mesenteroides to Acidified Brine

A laboratory test was conducted with some bacterial isolates in one of the previous tests and classified as *Leuconostoc mesenteroides*. The aim of the test was to evaluate bacterial survival in an acidified low-salt brine, free of the phenolic compounds released by the fruit and the spCO_2_ presence in the previous fermentation tests. Bacterial survival was evaluated in Pyrex tubes equipped with screw caps containing 9 mL of sterile non-acidified brine with pH 7.23 and brines with pH 2.30, acidified with 0.5% (*w v*^−1^) citric acid and 1N HCl solution, respectively. The brines contained 6% (*w v*^−1^) NaCl and were filtered through 0.45 µm nitrocellulose filters (Minisart NML-Sartorius, Göttingen, Germany) before being placed in Pyrex tubes previously sterilized at 120 °C for 20 min. Three strains of *L. mesenteroides* with similar physiological characteristics, designated as *L. mesenteroides* 2012, *L. mesenteroides* 2013, and *L. mesenteroides* 2014, were reproduced on NA medium for 48 h at 30 °C and used for the inoculation of the above-mentioned brines. The inoculum was prepared by transferring a portion of the bacterial biomass into three Pyrex tubes containing 9 mL of sterile Ringer’s solution. After shaking, the O.D._595_ of the three bacterial cultures was adjusted to 0.900 with another sterile solution. Finally, 1 mL of each bacterial suspension was transferred into Pyrex tubes with different brines and stored for 30 days at room temperature in a dark place. The test was accomplished with three repetitions. The survival of the three *L. mesenteroides* strains was assessed by analyzing 1 mL of each brine at the beginning and after 1, 2, 7, 15, and 30 days of incubation, using the same procedure described in the microbiological analysis of TAB.

### 2.8. Microbiological Analysis of Bactrocera Oleae Larvae

The *B. oleae* larvae free in the brine were collected and transferred to empty Petri dishes. After washing three times with sterile distilled water, the larvae were dried on paper towels before being weighed. Finally, they were transferred to a 25 mL beaker and homogenized with an Ultraturrax (IKA, Milan, Italy) for 1 min in the presence of sterile Ringer’s solution. The samples were serially diluted by a factor 10 in sterile Ringer’s solution and spread on specific agar medium as described before. The microbiological analysis was repeated three times.

### 2.9. Statistical Analysis

Statistical software was used for data processing (Statsoft version 7.0 for Windows, Tulsa, OK, USA). Comparisons among means performed using Duncan’s multiple-range tests (one-way ANOVA), and differences were considered significant at *p* < 0.05.

## 3. Results and Discussion

The traditional processing of black table olives involves several critical issues, including a high salt concentration, which helps control the growth of contaminating microorganisms. Both yeasts and bacteria are involved in the natural processing of black table olives. With the exception of some species, yeasts require less attention than bacteria. In fact, there are several unwanted contaminating bacteria, responsible for spoilage by producing off-flavors and toxins harmful to consumer health. Bacteria belonging to the *Clostridium* sp. deserve particular attention. This genus can metabolize lactic acid to produce butyric acid, leading to putrid odor and butyric fermentation. Secondary fermentation transforms lactic acid into other organic acids, which increases the pH of the product, making it more vulnerable to the growth of pathogenic microorganisms. A pH value ≥ 4.6 and NaCl concentration ≤ 10% (*w v*^−1^) enable the germination of *Cl. botulinum* spores, which are responsible for producing botulinum toxins, posing a health risk to consumers [7,28]. For these reasons, brine with pH < 4.3 and high concentrations of NaCl equal to 10–12% (*w v*^−1^) are used in the traditional natural fermentation of the black table olives. The “hurdle effect” induced by spCO_2_, which is associated with a low pH, especially at the beginning of fermentation, could balance the lesser protective action provided by the low-salt brine with 6% (*w v*^−1^) NaCl.

### 3.1. Fermentation with Healthy Olives

The fermentation test performed with washed healthy olives showed a reduced number of yeasts during the first days of incubation and the absence of bacteria and molds throughout the entire fermentation period under spCO_2_ conditions. The number of yeasts in the brine during the first 2 months of incubation varied from 2.81 to 4.85 Log CFU per mL, whereas it was almost constant in subsequent months. The prevalence of the yeast *Saccharomyces cerevisiae* varied from 89% to 100% in the brine throughout the fermentation period, whereas it reached 100% in the flesh at the end of the incubation period (Table 1). The absence of bacteria and mold, the lowest value of total yeasts recorded during the first 7 days of incubation, and the predominance of *S. cerevisiae* were in agreement with findings from other pilot models investigating the use of spCO_2_ to enhance low-salt brine antimicrobial activity [18]. The slow increase in the number of total yeasts recorded after the first week of incubation in brine subjected to spCO_2_ is attributable to the different resistance of microbial cells to adverse conditions through the use of baroprotective compounds [29]. The results concerning the predominance of *S. cerevisiae* were consistent with those of the studies cited above, thus confirming the ability of spCO_2_ to inhibit the growth of certain oxidizing yeasts in acidified low-salt brine and foster oleuropeinolytic and fermenting *S. cerevisiae*. This yeast species employs complex regulation strategies to tolerate low pH stress including the activation of proton pumps and transporters, metabolic adjustments, and changes in its cell membrane and structure [30]. The greater resilience of the fermenting yeast *S. cerevisiae* to spCO_2_, unlike many other non-fermenting species, is linked to its metabolism under anaerobic conditions that allow the alcoholic fermentation of sugars. The predominance of *S. cerevisiae* throughout the fermentation process could have positive effects on the aromatic profile of the olives. In a previous study, Leccino olives fermented under spCO_2_ conditions and subjected to sensory analysis showed a high score for the overall quality attribute [22].

The physico–chemical characteristics of the acidified (0.5%, *w v*^−1^, citric acid) low-salt (6%, *w v*^−1^, NaCl) brine indicated a slight increase in pH during the first few days of incubation because of the buffer power exercised by the components of the fruit. However, in the months following incubation, the pH values remained close to the hygienic safety threshold in all the samples analyzed [6]. The NaCl content in the brine increased slightly in the first 4 months, after which it stabilized. The flesh at the end of the incubation period contained 3.73% (*w v*^−1^) NaCl. These results confirm some of our previous data, which reported a 3.43% (*w v*^−1^) to 4.09% (*w v*^−1^) NaCl range in the flesh of olives processed with low-salt brine under spCO_2_ compared to 7.01% (*w v*^−1^) NaCl in that of olives processed traditionally with 11% (*w v*^−1^) NaCl [19]. The free CO_2_ content in the brine reached its maximum value at the end of the incubation. In contrast, the phenolic concentration of the brine reached the same value as that of the flesh after 6 months of incubation (Table 2).

The results of fermentation performed with unwashed, healthy olives often resembled those reported in fermentation with washed, healthy olives. Some noteworthy differences were observed in the microbiological analysis of the brine during incubation. One of the substantial differences compared to the results shown in Table 1 was the presence of molds in the brine recorded during first days of incubation (Table 3). This result may be related to the greater biodiversity of the initial microbiota in the samples from unwashed table olives. However, the presence of molds in the low-salt brine was only temporary as they disappeared after day 4 of incubation (Table 3). Referring to previous studies, we hypothesized that the disappearance of molds may be due to the action of spCO_2_, rather than acidic pH, which is favorable for molds [18]. The concentration of total yeasts, in this case, was low in the first 10 days of incubation and subsequently increased until it doubled at the end of the incubation. The heterogeneity of the original microbiota associated with the non-washing of healthy olives was highlighted by the appearance of molds in the brine and the presence of a greater number of yeast species (Table 3). Unlike fermentation with washed healthy table olives, where only two species prevailed at the beginning of incubation, in this case, more than four species of predominant yeasts were detected. *Candida boidinii*, *Kloeckera apiculata*, *Wickerhamomyces anomalus*, and *S. cerevisiae* were present in different proportions during the first 12 days of fermentation. A continuous increase in *S. cerevisiae* was observed during the rest of the fermentation, reaching 100% prevalence at the end of incubation. These results are in agreement with the data presented in Table 1 and with previous findings [19,22]. The physico–chemical analyses indicated a very low initial pH in the first 3 days of incubation, whereafter it gradually increased until it reached a maximum value of 3.94 at the end of the incubation. In synergy with spCO_2_, the low initial pH values produced by the addition of citric acid inhibited the growth of different microorganisms living in low-salt brine. The concentration of the free CO_2_ varied from 1.22 to 1.90 g kg^−1^ of brine (Table 4).

### 3.2. Fermentation with Damaged Olives

**Figure 1:**
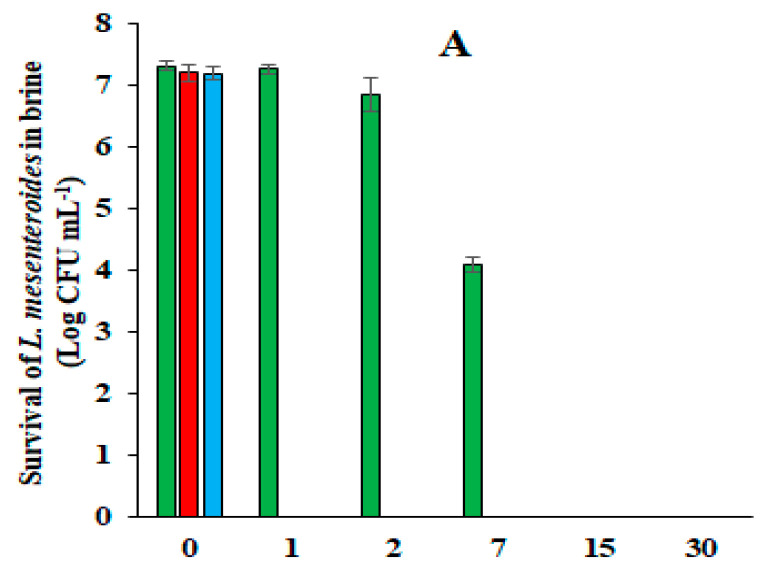

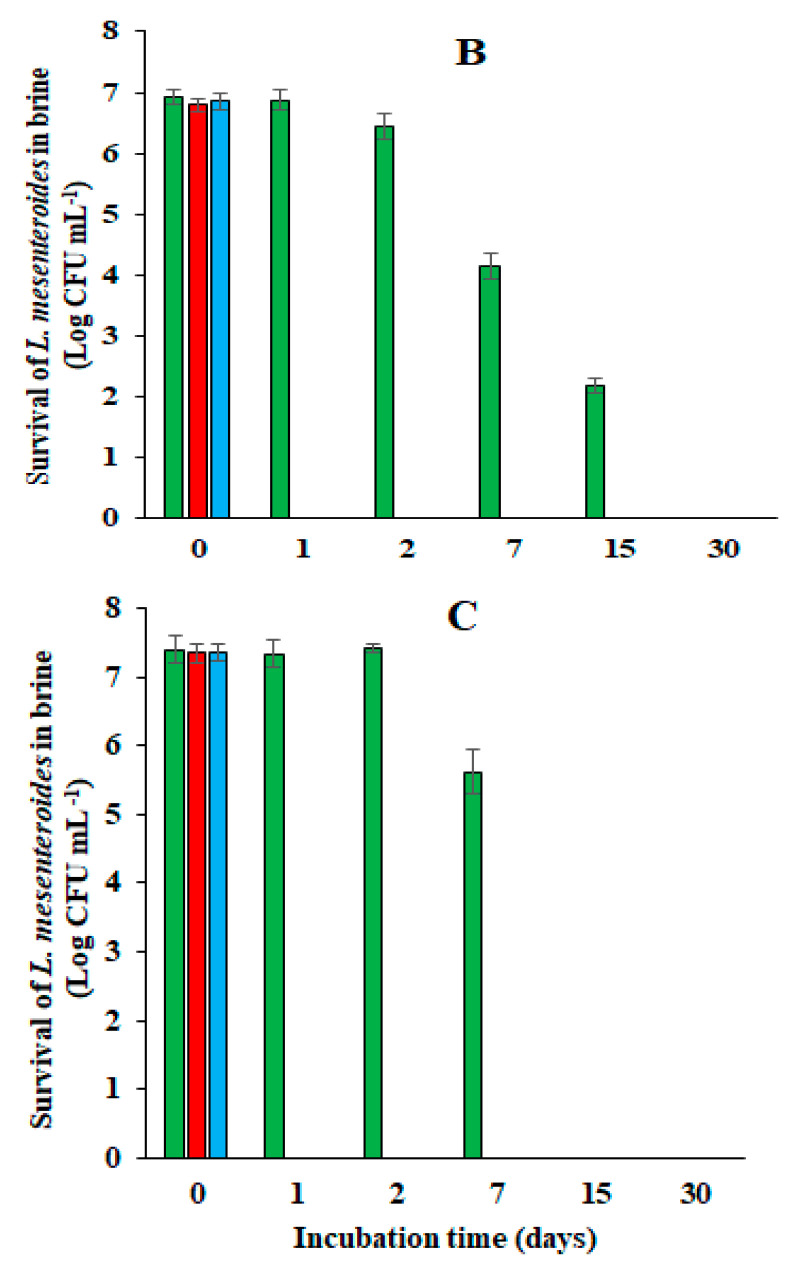
Survival of *L. mesenteroides* 2112 strain (**A**), 2113 strain (**B**), and 2114 strain (**C**) in low-salt brine (6%, *w v*^−1^) without olives, acidified with citric acid (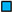), acidified with HCl (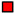), or not acidified (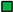).

The purpose of the third test was to study the antimicrobial activity of low-salt (6%, *w v*^−1^, NaCl) and acidified (0.5%, *w v*^−1^, citric acid) brine under spCO_2_ conditions characterized by low hygienic and sanitary requirements. The Leccino olives of the second batch were contaminated with several types of microorganisms and were damaged by *B. oleae* at an approximate rate of 10%. Furthermore, the low-salt brine was inoculated with a separately prepared brine aged for 7 days, which served as a starter culture at the beginning of the incubation (Table 5). The results of the microbiological analysis of the brine indicated the presence of a high number of yeasts from the beginning of the incubation period, the absence of molds, and the presence of a large number of TAB (Table 6). The total number of yeast cells increased slightly in the first days of incubation and then remained almost constant over the next 3 months until returning to the initial values at the end of the sixth month of incubation (Table 6). Compared to the other two previous tests, the high initial number of total yeasts can be attributed to the use of the inoculum, which was composed of approximately 80% *C. boidinii*. The yeast *C. boidinii* prevailed in the brine after inoculation only in the first days of incubation and was subsequently exceeded by *S. cerevisiae*, which reached 100% prevalence starting from the second month of incubation. The results concerning the affirmation of *S. cerevisiae* on other species are consistent because they confirm the data reported in Table 1 and Table 3 and previous findings [19,22]. However, unlike the other tests, a substantial number of TAB were recorded in the brine of the olives damaged by the fruit fly. This remained unchanged in the first week of incubation and then increased in the following 20 days, before it decreased and disappeared after 2 months of incubation (Table 6). Subsequent laboratory analyses confirmed that the TAB comprised a single type of bacterium identified as *Leuconostoc mesenteroides* whose morpho-physiological characteristics are reported in Table 7.This result seems unusual, it did not appear in previous tests performed with healthy olives, i.e., undamaged by fruit flies. Based on other laboratory tests, the survival of *L. mesenteroides* may depend on the initial pH of the brine. In fact, the initial pH of brine was 2.25 in fermentation with unwashed healthy olives; however, in this case, the addition of external brine as a starter increased the initial pH to 3.71 (Table 8). Furthermore, since the lactic acid bacterium *L. mesenteroides* grew in the first 2 months of incubation, it may have been favored by the greater availability of sugars from the fruit, as well as vitamins, amino acids, and purines produced by the abundant yeast population [31]. Although fermentation of this type of olive occurs under precarious hygienic and sanitary conditions, the antimicrobial activity of low-salt brine under spCO_2_ conditions was also confirmed in this test. In fact, all microorganisms disappeared after 2 months of incubation, with the exception of yeasts, with the predominance of *S. cerevisiae* (Table 6). The pH values recorded in the brine during incubation were slightly higher than those shown during the fermentation with healthy olives, starting from the first day of Incubation (Table 8). This trend could be linked to the greater buffering activity of the compounds released by the galleries of fruit damaged by the fly. Unlike the results shown in Table 2 and Table 4, Table 8 shows a greater increase in phenolic compounds during the 6 months of incubation. The dynamics of pH and phenolic content may have played important roles in both the appearance and disappearance of *L. mesenteroides* in low-salt brine during fermentation under spCO_2_ conditions. In fact, while the initial pH, being not excessively acid, may not have inhibited the growth of *L. mesenteroides*, the accumulation of phenols in the brine could have exerted a “hurdle effect” on the survival of the bacterium, ultimately leading to its death (Table 6). Regarding the source of the bacterial contamination in the brine, the precarious hygienic and sanitary conditions, the low integrity of the fruits, and the presence of microbiota associated with the larvae of *B. oleae* must be considered. Microbiological analyses of the free larvae of *B. oleae* collected from the brine showed a large number of enterobacteria and TAB (Table 9). Considering that *L. mesenteroides* was re-isolated from the samples of low-salt brine using the NA medium used precisely to count the TAB, the hypothesis that the bacterium *L. mesenteroides* originated from this bacterial group, highlighted in the larvae of *B. oleae*, becomes plausible. However, the enterobacteria, highlighted in the fruit fly larvae, did not survive in low-salt brine during fermentation under spCO_2_ conditions. *L. mesenteroides* is an oleuropeinolytic lactic bacterium that produces β-glucosidase and is commonly found in the brine of the naturally processed black table olives. Its role in natural fermentation seems more useful for the flavor of the olives rather than for debittering, since its β-glucosidase enzyme has an optimal pH of 8; therefore, it is strongly inhibited by the acidic pH of the brine [26]. An acidic pH also negatively impacts the survival of bacteria in low-salt brine. Laboratory tests showed that three strains of *L. mesenteroides* survived for less than 24 h in acidified brine free of olives, with 6% (*w v*^−1^) NaCl at pH 2.30. In contrast, a survival of 7–15 days was recorded in the same non-acidified brine (pH 7.23), depending on the strain used (Figure 1A–C). The short survival of the tested strains recorded in the brine without nutrients, equal to a maximum of 15 days, explains the higher survival of 2 months recorded in the low-salt brines of the olives damaged by *B. oleae*.

## 4. Conclusions

Olive processing remains a craft-based industry and has not undergone substantial changes since antiquity, resulting in a final product with unstable properties and potential health risk concerns, particularly due to the high salt content. Fermentation in the presence of spCO_2_ is a new olive processing method that, in contrast to the traditional method, can control the growth of spoilage microorganisms in brine with low salt content. The results of this study confirmed the antimicrobial activity of low-salt brine acidified with citric acid during the natural fermentation of black table olives under spCO_2_. The growth of bacteria and molds was controlled in the brine of healthy olives, which favored the growth of yeasts, including *S. cerevisiae*. The same inhibitory effect was observed when Leccino olives were severely damaged by the olive fruit fly, *B. oleae*. In this brine, characterized by severe hygiene deficiencies, enterobacteria were successfully controlled, while other bacterial species, including *L. mesenteroides* disappeared after 2 months of incubation, and yeasts, especially *S. cerevisiae*, prevailed during the incubation period. Based on these findings, the use of spCO_2_ may represent a new and promising approach for improving product quality by reducing the NaCl content in brine and fruits. Simultaneously, it reduces defects related to spoilage microorganisms, prevents the formation of microbial biofilms on the surface of brines, and ultimately reduces pollution caused by salt-rich wastewater and CO_2_ emissions into the atmosphere. However, further technological studies are required to investigate how this new processing system, applied to small and medium-sized commercial plastic containers with hermetic seals, can be transferred onto a large industrial scale. The seal system of the current 160–220 L PVC barrels and 10–20 ton stainless steel silos available on the market must be modified to withstand spCO_2_.

## Figures and Tables

**Table 1:** Microbiological analysis of washed healthy black table olives during natural fermentation with low-salt brine under spCO_2_.

Days	Total Yeasts (Log CFU mL^−1^ or g^−1^)		Preeminent Yeast Species (%)		Other Microorganisms (Log CFU mL^−1^ or g^−1^)	
	Brine	Flesh	Brine	Flesh	Brine	Flesh
7	2.81 ± 0.13 ^a^	n.d.	*S.c.* (100)	n.d.	<1 ^1^	<1
60	4.85 ± 0.24 ^b^	n.d.	*S.c.* (100)	n.d.	<1	<1
120	5.59 ± 0.15 ^b^	n.d.	*S.c.* (98) *C.b.* (2)	n.d.	<1	<1
180	5.99 ± 0.26 ^b^	n.d.	*S.c.* (94) *C.b.* (6)	n.d.	<1	<1
240	4.65 ± 0.12 ^b^	4.48 ± 0.18	*S.c.* (89) *C.b.* (11)	*S.c.* (100)	<1	<1

n.d., not determined. ^1^ <1, below the detection limit of 10 CFU mL^−1^ or g^−1^. *S.c.*, *Saccharomyces cerevisiae*; *C.b.*, *Candida boidinii*. Mean ± standard deviation (n. repetitions = 3). Values in columns with different letters are significantly different from each other at *p* < 0.05.

**Table 2:** Physicochemical parameters of washed, healthy black table olives during natural fermentation under low-salt brine and spCO_2_ conditions.

Days	pH		NaCl (%, *w v*^−1^ or *w*^−1^)		Free CO_2_ (g kg^−1^)		Total Polar Phenols (mg CAE mL^−1^ or g^−1^)	
	Brine	Flesh	Brine	Flesh	Brine	Flesh	Brine	Flesh
7	3.44 ± 0.06	n.d.	3.50 ± 0.10 ^a^	n.d.	1.40 ± 0.20 ^a^	n.d.	1.20 ± 0.10 ^a^	5.00 ± 0.10 ^d^
60	4.02 ± 0.06	n.d.	3.75 ± 0.10 ^a^	n.d.	1.70 ± 0.19 ^a^	n.d.	1.43 ± 0.21 ^a^	4.52 ± 0.09 ^c^
120	4.04 ± 0.04	n.d.	4.05 ± 0.04 ^ab^	n.d.	1.89 ± 0.02 ^ab^	n.d.	2.03 ± 0.00 ^b^	3.61 ± 0.08 ^b^
180	4.24 ± 0.02	n.d.	4.95 ± 0.02 ^b^	n.d.	1.96 ± 0.06 ^ab^	n.d.	2.61 ± 0.12 ^c^	2.61 ± 0.00 ^a^
240	4.32 ± 0.09	4.50 ± 0.08	4.40 ± 0.10 ^b^	3.73 ± 0.15	2.64 ± 0.01 ^b^	n.d.	2.79 ± 0.07 ^c^	2.79 ± 0.02 ^a^

n.d., not determined. CAE, caffeic acid equivalent. Mean ± standard deviation (n. repetitions = 3). Values in columns with different letters are significantly different from each other at *p* < 0.05.

**Table 3:** Microbiological analysis of unwashed healthy black table olives during natural fermentation under low-salt brine and spCO_2_ conditions.

Days	Total Yeasts (Log CFU mL^−1^)	Preeminent Yeast Species (%)	Total Molds (Log CFU mL^−1^)	Others ^1^
1	2.68 ± 0.08 ^a^	*W.a.* (89) Other (11)	2.51 ± 0.20	<1 ^2^
2	2.19 ± 0.15 ^a^	*K.a.* (64) *W.a.* (36)	3.33 ± 0.38	<1
3	2.31 ± 0.11 ^a^	*K.a.* (50) *W.a.* (40) *C.b.* (10)	2.63 ± 0.24	<1
4	2.44 ± 0.37 ^a^	*C.b.* (50) Other (50)	<1	<1
5	2.15 ± 0.17 ^a^	*C.b.* (70) Other (30)	<1	<1
6	2.10 ± 0.15 ^a^	*C.b.* (75) Other (25)	<1	<1
7	1.90 ± 0.20 ^a^	n.d.	<1	<1
8	1.85 ± 0.21 ^a^	n.d.	<1	<1
9	1.90 ± 0.19 ^a^	n.d.	<1	<1
10	1.89 ± 0.13 ^a^	n.d.	<1	<1
11	3.52 ± 0.92 ^b^	*C.b.* (98) *S.c.* (2)	<1	<1
12	3.50 ± 0.19 ^b^	*S.c.* (70) *C.b.* (30)	<1	<1
13	3.48 ± 0.10 ^b^	*S.c.* (75) *C.b.* (25)	<1	<1
14	3.40 ± 0.11 ^b^	*S.c.* (77) *C.b.* (23)	<1	<1
15	3.42 ± 0.20 ^b^	*S.c.* (80) *C.b.* (20)	<1	<1
16	3.43 ± 0.19 ^b^	*S.c.* (98) *C.b.* (2)	<1	<1
17	3.37 ± 0.15 ^b^	*S.c.* (100)	<1	<1
18	4.17 ± 0.26 ^c^	*S.c.* (100)	<1	<1
19	4.49 ± 0.23 ^c^	*S.c.* (100)	<1	<1
20	4.10 ± 0.10 ^c^	*S.c.* (100)	<1	<1
21	4.30 ± 0.20 ^c^	*S.c.* (100)	<1	<1
22	4.17 ± 0.18 ^c^	*S.c.* (100)	<1	<1
23	4.44 ± 0.22 ^c^	*S.c.* (100)	<1	<1
24	4.27 ± 0.93 ^c^	*S.c.* (98) *C.b.* (2)	<1	<1
25	4.48 ± 0.15 ^c^	*S.c.* (100)	<1	<1

^1^ Total aerobic, anaerobic, and enterobacterial communities. ^2^ <1, below the detection limit of 10 CFU mL^−1^. Mean ± standard deviation (n. repetitions = 3). *W.a.*, *Wickerhamomyces anomalus*; *K.a.*, *Kloeckera apiculata*; *C.b.*, *Candida boidinii*; *S.c.*, *Saccharomyces cerevisiae*; n.d., not detected. For brine samples where the total yeasts values were <2, the yeast colonies were not used for the preparation of master cultures for species identification. Values in columns with different letters are significantly different from each other at *p* < 0.05.

**Table 4:** Physico–chemical analysis of unwashed, healthy black table olives during natural fermentation under low-salt brine and spCO_2_ conditions.

Days	pH	Free CO_2_ (g kg^−1^)	Total Polar Phenols (mg CAE mL^−1^)
1	2.25 ± 0.09 ^a^	1.22 ± 0.09 ^a^	0.09 ± 0.00 ^a^
2	2.32 ± 0.04 ^a^	1.26 ± 0.07 ^a^	0.17 ± 0.02 ^a^
3	2.42 ± 0.03 ^a^	1.29 ± 0.05 ^a^	0.33 ± 0.05 ^a^
4	2.56 ± 0.08 ^ab^	1.39 ± 0.09 ^a^	0.37 ± 0.03 ^a^
5	2.65 ± 0.06 ^ab^	1.48 ± 0.10 ^a^	0.49 ± 0.02 ^a^
6	3.00 ± 0.06 ^b^	1.48 ± 0.11 ^a^	0.57 ± 0.06 ^a^
7	3.06 ± 0.07 ^b^	1.49 ±0.06 ^a^	0.59 ± 0.07 ^ab^
8	3.06 ± 0.06 ^b^	1.49 ± 0.25 ^a^	0.69 ± 0.05 ^ab^
9	3.07 ± 0.01 ^b^	1.54 ± 0.12 ^a^	0.98 ± 0.08 ^b^
10	3.17 ± 0.05 ^b^	1.59 ± 0.06 ^a^	1.08 ± 0.05 ^b^
11	3.31 ± 0.01 ^bc^	1.62 ± 0.07 ^ab^	1.15 ± 0.06 ^b^
12	3.37 ± 0.02 ^bc^	1.70 ± 0.03 ^ab^	1.38 ± 0.01 ^b^
13	3.37 ± 0.03 ^bc^	1.72 ± 0.06 ^ab^	1.50 ± 0.07 ^b^
14	3.40 ± 0.02 ^bc^	1.76 ± 0.08 ^ab^	1.66 ± 0.08 ^b^
15	3.45 ± 0.04 ^bc^	1.80 ± 0.04 ^ab^	1.72 ± 0.03 ^bc^
16	3.50 ± 0.13 ^c^	1.82 ± 0.03 ^ab^	1.88 ± 0.05 ^bc^
17	3.74 ± 0.09 ^c^	1.84 ± 0.11 ^b^	1.88 ± 0.01 ^bc^
18	3.78 ± 0.00 ^c^	1.85 ± 0.09 ^b^	1.89 ± 0.00 ^bc^
19	3.80 ± 0.03 ^c^	1.88 ± 0.06 ^b^	1.91 ± 0.02 ^c^
20	3.84 ± 0.01 ^c^	1.88 ± 0.05 ^b^	1.92 ± 0.05 ^c^
21	3.86 ± 0.07 ^cd^	1.89 ± 0.09 ^b^	1.94 ± 0.01 ^c^
22	3.88 ± 0.06 ^cd^	1.90 ± 0.13 ^b^	1.94 ± 0.07 ^c^
23	3.89 ± 0.04 ^cd^	1.85 ± 0.05 ^b^	1.96 ± 0.06 ^c^
24	3.91 ± 0.05 ^d^	1.80 ± 0.11 ^ab^	1.98 ± 0.02 ^c^
25	3.94 ± 0.04 ^d^	1.74 ± 0.03 ^ab^	1.99 ± 0.03 ^c^

CAE, caffeic acid equivalent. Mean ± standard deviation (n. repetitions = 3). Values in columns with different letters are significantly different from each other at *p* < 0.05.

**Table 5:** Microbiological characteristics of the fermented brine used as the starter.

Total Yeasts (Log CFU mL^−1^)	Preeminent Yeast Species (%)	Total Molds (Log CFU mL^−1^)	TAB (Log CFU mL^−1^)	TANB (Log CFU mL^−1^)	Enterobacteria (Log CFU mL^−1^)
6.31 ± 0.16	*C.b.* (80) Others (20)	<1 ^1^	<1	<1	<1

^1^ <1, below the detection limit of 10 CFU mL^−1^. Mean ± standard deviation (n. repetitions = 3). *C.b.*, *Candida boidinii*. TAB, Total Aerobic Bacteria; TANB, Total Anaerobic Bacteria.

**Table 6:** Microbiological analysis of damaged and unwashed black table olives during natural fermentation under low-salt brine and spCO_2_ conditions.

Days	Total Yeasts (Log CFU mL^−1^)	Preeminent Yeast Species (%)	Total Molds (Log CFU mL^−1^)	TAB (Log CFU mL^−1^)	Other Bacteria ^1^
3	5.10 ± 0.16 ^a^	*C.b.* (76) *S.c.* (22) *W.a.* (2)	<1 ^2^	4.66 ± 0.50 ^a^	<1
4	5.66 ± 0.12 ^ab^	*S.c.* (58) *C.b.* (42)	<1	4.70 ± 0.23 ^a^	<1
5	6.07 ± 0.08 ^b^	*S.c.* (84) *C.b.* (16)	<1	4.74 ± 0.64 ^a^	<1
6	6.21 ± 0.17 ^b^	*S.c.* (98) *C.b.* (2)	<1	4.88 ± 0.55 ^ab^	<1
9	6.44 ± 0.21 ^b^	*S.c.* (86) *C.b.* (14)	<1	5.63 ± 0.53 ^b^	<1
10	6.29 ± 0.19 ^b^	*S.c.* (90) *C.b.* (10)	<1	5.33 ± 0.83 ^b^	<1
11	6.19 ± 0.13 ^b^	*S.c.* (90) *C.b.* (10)	<1	5.72 ± 0.18 ^b^	<1
12	6.15 ± 0.27 ^b^	*S.c.* (98) *C.b.* (2)	<1	5.08 ± 0.47 ^b^	<1
16	6.26 ± 0.26 ^b^	*S.c.* (100)	<1	5.60 ± 0.33 ^b^	<1
18	6.37 ± 0.07 ^b^	*S.c.* (100)	<1	5.74 ± 0.17 ^b^	<1
20	6.48 ± 0.11 ^b^	*S.c.* (98) *C.b.* (2)	<1	4.40 ± 0.12 ^a^	<1
23	6.55 ± 0.11 ^b^	*S.c.* (98) *C.b.* (2)	<1	4.09 ± 0.12 ^a^	<1
50	6.31 ± 0.18 ^b^	*S.c.* (100)	<1	4.77 ± 0.36 ^a^	<1
57	6.24 ± 0.09 ^b^	*S.c.* (100)	<1	4.22 ± 0.91 ^a^	<1
64	6.33 ± 0.06 ^b^	*S.c.* (92) *C.b.* (8)	<1	<1	<1
71	5.98 ± 0.27 ^ab^	*S.c.* (100)	<1	<1	<1
85	5.75 ± 0.26 ^ab^	*S.c.* (100)	<1	<1	<1
180	5.48 ± 0.20 ^a^	*S.c.* (100)	<1	<1	<1

^1^ Total anaerobic bacteria, and enterobacteria. TAB, total aerobic bacteria. ^2^ <1, below the detection limit of 10 CFU mL^−1^. Mean ± standard deviation (n. repetitions = 3). *C.b.*, *Candida boidinii*; *S.c.*, *Saccharomyces cerevisiae*; *W.a.*, *Wickerhamomyces anomalus*. Values in columns with different letters are significantly different from each other at *p* < 0.05.

**Table 7:** Morphological and physiological characteristics of *Leuconostoc* *mesenteroides* isolated from low-salt brine under spCO_2_ conditions.

Tests	Observations
Shape	Coccus
Gram’s stain	+
Growth at 45 °C	−
Growth at 15 °C	+
Growth with 4.0% (*w v*^−1^) NaCl	+
Growth with 6.5% (*w v*^−1^) NaCl	−
Catalase test	−
Gas from glucose	+
NH_3_ production	−
NO_3_ reduction	−
Dextran production	+
Litmus milk coagulation	+
Fermentation of:	
maltose	+
fructose	+
trehalose	+
raffinose	+/−
lactose	+/−
arabinose	+

+, positive reaction; −, negative reaction; +/−, delayed positive.

**Table 8:** Physico–chemical parameters of the damaged black table olives during natural fermentation under low-salt brine and spCO_2_ conditions.

Days	pH	Free CO_2_ (g kg^−1^)	Total Polar Phenols (mg CAE mL^−1^)
1	3.71 ± 0.05 ^a^	1.62 ± 0.14 ^a^	0.11 ± 0.02 ^a^
2	3.75 ± 0.01 ^a^	1.67 ± 0.16 ^a^	0.20 ± 0.08 ^a^
3	3.79 ± 0.04 ^a^	1.71 ± 0.12 ^a^	0.36 ± 0.07 ^a^
4	3.83 ± 0.10 ^a^	1.79 ± 0.03 ^a^	0.40 ± 0.01 ^a^
5	3.92 ± 0.04 ^ab^	1.88 ± 0.21 ^b^	0.52 ± 0.05 ^ab^
6	3.82 ± 0.03 ^a^	1.92 ± 0.07 ^b^	0.60 ± 0.03 ^ab^
9	3.92 ± 0.00 ^ab^	2.02 ± 0.03 ^b^	1.08 ± 0.06 ^b^
10	3.89 ± 0.04 ^ab^	1.99 ± 0.13 ^b^	1.12 ± 0.09 ^b^
11	3.82 ± 0.01 ^a^	1.97 ± 0.08 ^b^	1.20 ± 0.05 ^b^
12	3.88 ± 0.04 ^ab^	1.95 ± 0.07 ^b^	1.42 ± 0.04 ^b^
16	4.03 ± 0.12 ^b^	1.96 ± 0.13 ^b^	1.91 ± 0.04 ^bc^
18	4.08 ± 0.10 ^b^	1.84 ± 0.11 ^b^	1.93 ± 0.06 ^bc^
20	4.10 ± 0.09 ^b^	1.72 ± 0.09 ^a^	1.95 ± 0.01 ^bc^
23	4.16 ± 0.06 ^b^	1.68 ± 0.23 ^a^	1.98 ± 0.05 ^bc^
50	4.44 ± 0.01 ^b^	1.72 ± 0.48 ^a^	2.25 ± 0.10 ^c^
57	4.44 ± 0.02 ^b^	1.79 ± 0.23 ^a^	2.32 ± 0.09 ^c^
64	4.46 ± 0.03 ^b^	1.91 ± 0.15 ^b^	2.39 ± 0.11 ^c^
71	4.47 ± 0.05 ^b^	1.86 ± 0.08 ^bc^	2.46 ± 0.01 ^cd^
85	4.47 ± 0.08 ^b^	1.84 ± 0.16 ^bc^	3.02 ± 0.11 ^d^
180	4.47 ± 0.12 ^b^	1.80 ± 0.13 ^a^	3.57 ± 0.08 ^d^

CAE, caffeic acid equivalent. Mean ± standard deviation (n. repetitions = 3). Values in columns with different letters are significantly different from each other at *p* < 0.05.

**Table 9:** Microbiological analysis of *Bactrocera oleae* larvae collected from low-salt brine during fermentation of damaged black table olives.

Total Yeasts (Log CFU g^−1^)	Total Molds (Log CFU g^−1^)	Enterobacteria (Log CFU g^−1^)	TAB (Log CFU g^−1^)	TANB (Log CFU g^−1^)
<1 ^1^	<1	6.53 ± 0.16	8.02 ± 0.26	<1

^1^ <1, below the detection limit of 10 CFU g^−1^. Mean ± standard deviation (n. repetitions = 3). TAB, total aerobic bacteria; TANB, total anaerobic bacteria.

## Data Availability

The original contributions presented in this study are included in the article, and further inquiries can be directed to the corresponding author.
